# Supplementary material for: Multi‐dataset identification of innovative feature genes and molecular mechanisms in keratoconus
Source: J Cell Mol Med. 2024 Sep 19;28(18):e70079. doi: 10.1111/jcmm.70079 (PMC11412914; doi:10.1111/jcmm.70079)
Supplement: Supplementary file 2 — Material S2. [file JCMM-28-e70079-s002.zip › 7.GSEA analysis/5.RNF217-AS1/KEGG_GAP_JUNCTION.html]

Details for gene set KEGG\_GAP\_JUNCTION[GSEA]

|  || Dataset | RNF217-AS1.group.cls#h\_versus\_l |
| Phenotype | group.cls#h\_versus\_l |
| Upregulated in class | h |
| GeneSet | KEGG\_GAP\_JUNCTION |
| Enrichment Score (ES) | 0.16720694 |
| Normalized Enrichment Score (NES) | 0.75529283 |
| Nominal p-value | 0.94716984 |
| FDR q-value | 1.0 |
| FWER p-Value | 1.0 |
Table: GSEA Results Summary

  

Fig 1: Enrichment plot: KEGG\_GAP\_JUNCTION      
 Profile of the Running ES Score & Positions of GeneSet Members on the Rank Ordered List

  

| SYMBOL | TITLE | RANK IN GENE LIST | RANK METRIC SCORE | RUNNING ES | CORE ENRICHMENT || 1 | PRKCG | na | 203 | 0.657 | 0.0563 | Yes |
| 2 | GUCY1A2 | na | 1454 | 0.397 | 0.0578 | Yes |
| 3 | HTR2C | na | 2142 | 0.339 | 0.0700 | Yes |
| 4 | TUBB1 | na | 2778 | 0.301 | 0.0802 | Yes |
| 5 | ADCY2 | na | 2970 | 0.293 | 0.1024 | Yes |
| 6 | GRM5 | na | 3284 | 0.279 | 0.1197 | Yes |
| 7 | ADCY4 | na | 4384 | 0.238 | 0.1105 | Yes |
| 8 | ADCY5 | na | 4777 | 0.225 | 0.1205 | Yes |
| 9 | TUBB4A | na | 5303 | 0.206 | 0.1248 | Yes |
| 10 | ADCY7 | na | 5579 | 0.197 | 0.1355 | Yes |
| 11 | TUBA3D | na | 5668 | 0.194 | 0.1512 | Yes |
| 12 | PDGFB | na | 6818 | 0.158 | 0.1331 | Yes |
| 13 | GNAQ | na | 7033 | 0.152 | 0.1413 | Yes |
| 14 | TUBA8 | na | 7048 | 0.151 | 0.1552 | Yes |
| 15 | PLCB3 | na | 7444 | 0.141 | 0.1571 | Yes |
| 16 | GNAI3 | na | 7550 | 0.139 | 0.1672 | Yes |
| 17 | PRKG1 | na | 8062 | 0.126 | 0.1644 | No |
| 18 | SOS1 | na | 9467 | 0.096 | 0.1329 | No |
| 19 | TUBA4A | na | 9754 | 0.090 | 0.1332 | No |
| 20 | PRKG2 | na | 10584 | 0.077 | 0.1165 | No |
| 21 | ADCY1 | na | 10624 | 0.076 | 0.1226 | No |
| 22 | TUBB6 | na | 10934 | 0.071 | 0.1204 | No |
| 23 | KRAS | na | 11445 | 0.064 | 0.1117 | No |
| 24 | PDGFA | na | 12021 | 0.055 | 0.1003 | No |
| 25 | TJP1 | na | 12076 | 0.054 | 0.1039 | No |
| 26 | TUBB3 | na | 12169 | 0.053 | 0.1062 | No |
| 27 | ITPR3 | na | 12500 | 0.049 | 0.1013 | No |
| 28 | HTR2B | na | 12943 | 0.043 | 0.0926 | No |
| 29 | TUBA3C | na | 13191 | 0.040 | 0.0892 | No |
| 30 | MAPK1 | na | 13362 | 0.038 | 0.0879 | No |
| 31 | PDGFRB | na | 13387 | 0.038 | 0.0908 | No |
| 32 | SRC | na | 13502 | 0.036 | 0.0909 | No |
| 33 | EGF | na | 13646 | 0.034 | 0.0900 | No |
| 34 | MAPK3 | na | 14127 | 0.029 | 0.0789 | No |
| 35 | MAP3K2 | na | 14371 | 0.026 | 0.0744 | No |
| 36 | TUBB4B | na | 14492 | 0.025 | 0.0733 | No |
| 37 | GNAI2 | na | 14991 | 0.019 | 0.0607 | No |
| 38 | CSNK1D | na | 15013 | 0.019 | 0.0619 | No |
| 39 | HRAS | na | 15878 | 0.010 | 0.0379 | No |
| 40 | MAP2K2 | na | 16236 | 0.006 | 0.0282 | No |
| 41 | GNA11 | na | 16472 | 0.004 | 0.0218 | No |
| 42 | MAPK7 | na | 16971 | -0.001 | 0.0076 | No |
| 43 | ADCY9 | na | 17206 | -0.004 | 0.0011 | No |
| 44 | PDGFRA | na | 17252 | -0.004 | 0.0002 | No |
| 45 | NRAS | na | 17304 | -0.005 | -0.0008 | No |
| 46 | GNAS | na | 17807 | -0.009 | -0.0144 | No |
| 47 | TUBB | na | 18395 | -0.014 | -0.0300 | No |
| 48 | RAF1 | na | 18969 | -0.020 | -0.0447 | No |
| 49 | ADCY6 | na | 19355 | -0.024 | -0.0536 | No |
| 50 | SOS2 | na | 19710 | -0.027 | -0.0612 | No |
| 51 | TUBA1C | na | 19897 | -0.029 | -0.0638 | No |
| 52 | CDK1 | na | 20745 | -0.037 | -0.0848 | No |
| 53 | TUBB2A | na | 20999 | -0.040 | -0.0883 | No |
| 54 | PLCB4 | na | 21374 | -0.044 | -0.0949 | No |
| 55 | ADRB1 | na | 21391 | -0.044 | -0.0912 | No |
| 56 | ADCY8 | na | 21903 | -0.050 | -0.1013 | No |
| 57 | ITPR2 | na | 21984 | -0.051 | -0.0988 | No |
| 58 | GRB2 | na | 22374 | -0.055 | -0.1049 | No |
| 59 | DRD1 | na | 22536 | -0.056 | -0.1042 | No |
| 60 | ADCY3 | na | 22794 | -0.059 | -0.1060 | No |
| 61 | PRKACA | na | 23066 | -0.063 | -0.1079 | No |
| 62 | MAP2K1 | na | 23215 | -0.064 | -0.1061 | No |
| 63 | GJD2 | na | 23350 | -0.066 | -0.1037 | No |
| 64 | PRKCA | na | 23805 | -0.072 | -0.1099 | No |
| 65 | TUBB8 | na | 24137 | -0.077 | -0.1122 | No |
| 66 | EGFR | na | 24191 | -0.078 | -0.1063 | No |
| 67 | TUBA1B | na | 24434 | -0.082 | -0.1056 | No |
| 68 | GJA1 | na | 24611 | -0.084 | -0.1027 | No |
| 69 | ITPR1 | na | 25459 | -0.098 | -0.1179 | No |
| 70 | MAP2K5 | na | 25571 | -0.100 | -0.1117 | No |
| 71 | TUBA1A | na | 25638 | -0.101 | -0.1041 | No |
| 72 | LPAR1 | na | 25719 | -0.102 | -0.0968 | No |
| 73 | TUBB2B | na | 25992 | -0.107 | -0.0945 | No |
| 74 | PRKACB | na | 26457 | -0.116 | -0.0970 | No |
| 75 | DRD2 | na | 26679 | -0.119 | -0.0920 | No |
| 76 | GRM1 | na | 28181 | -0.153 | -0.1209 | No |
| 77 | HTR2A | na | 28374 | -0.158 | -0.1115 | No |
| 78 | GNAI1 | na | 28406 | -0.159 | -0.0973 | No |
| 79 | PDGFC | na | 28424 | -0.160 | -0.0827 | No |
| 80 | PLCB2 | na | 31149 | -0.248 | -0.1379 | No |
| 81 | PRKX | na | 31362 | -0.257 | -0.1197 | No |
| 82 | PLCB1 | na | 32371 | -0.315 | -0.1190 | No |
| 83 | TUBAL3 | na | 32996 | -0.360 | -0.1030 | No |
| 84 | PRKACG | na | 33174 | -0.374 | -0.0728 | No |
| 85 | PRKCB | na | 34175 | -0.520 | -0.0524 | No |
| 86 | PDGFD | na | 34588 | -0.723 | 0.0040 | No |
Table: GSEA details [plain text format]

  

Fig 2: KEGG\_GAP\_JUNCTION      
 Blue-Pink O' Gram in the Space of the Analyzed GeneSet

  

Fig 3: KEGG\_GAP\_JUNCTION: Random ES distribution      
 Gene set null distribution of ES for **KEGG\_GAP\_JUNCTION**
